# Process Analysis of Millet Bran Fermentation by *Bacillus natto*: Dynamic Changes in Enzyme Activities and Bioactive Components

**DOI:** 10.3390/foods15030483

**Published:** 2026-01-30

**Authors:** Shimei Zhang, Fanqiang Meng, Xia Fan, Fengxia Lv, Xiaomei Bie, Haizhen Zhao

**Affiliations:** College of Food Science and Technology, Nanjing Agricultural University, Nanjing 210095, China; 2021108069@stu.njau.edu.cn (S.Z.); mfq@njau.edu.cn (F.M.); fanxia@njau.edu.cn (X.F.); lufengxia@njau.edu.cn (F.L.); bxm43@njau.edu.cn (X.B.)

**Keywords:** millet bran, *Bacillus natto*, fermentation time, enzyme activity, bioactive components

## Abstract

To investigate the dynamic changes during millet bran fermentation by *Bacillus natto*, we systematically monitored microbial growth, key enzyme activities, and the contents of major bioactive components over time. The changes in viable bacterial count, spore count, and the activities of protease, amylase, cellulase, and nattokinase (NK) fibrinolytic activity were measured throughout the 0–84 h fermentation process. Concurrently, variations in the contents of total sugars, reducing sugars, soluble dietary fiber (SDF), β-glucan, arabinoxylan, peptides, and polyphenols were analyzed. The results indicated that the viable bacterial count in the fermentation broth peaked at 48 h (9.3 log CFU/mL) and subsequently declined, while the spore count significantly increased to 7.6 log CFU/mL by 84 h. The activities of protease, amylase, cellulase, and NK fibrinolytic activity all exhibited a trend of initial increase followed by a decrease, reaching their respective maximum levels at 48 h. The contents of SDF, peptides, and polyphenols attained their highest values at 60 h, corresponding to 2.4 times, 2.17 times, and 1.5 times those of the unfermented control, respectively. The β-glucan content peaked at 24 h (31.31 mg/g millet bran), whereas the arabinoxylan content reached its maximum at 60 h, which was 19.4 times higher than that of the unfermented sample. Based on a comprehensive evaluation of all indicators, 48–60 h was determined to be the optimal fermentation duration for millet bran using *B. natto*. This research elucidates the relationship between enzyme activities and the accumulation of active components during fermentation, providing a theoretical foundation for the high-value utilization of millet bran and the development of functional products.

## 1. Introduction

Millet bran (MB), a significant by-product of millet processing, is rich in active substances including dietary fibers, phenolics, flavonoids, and antioxidants, with potential for functional food use [1]. Despite its demonstrated health benefits such as antioxidant and prebiotic properties, the utilization of millet bran in the food industry remains limited due to several factors: its coarse texture, poor solubility, the presence of anti-nutritional factors that hinder nutrient bioavailability, and limited public awareness of its bioactive potential. Currently, the majority of millet bran is utilized as animal feed, representing suboptimal resource utilization efficiency.

Microbial fermentation has emerged as a promising strategy to enhance the added value of agricultural by-products by modifying their structural and functional properties. Among various microorganisms, *Bacillus subtilis* is widely employed in food fermentation due to its ability to produce a diverse array of extracellular enzymes, including proteases, amylases, cellulases, and nattokinase (NK) [2,3,4,5]. These enzymes play a crucial role in breaking down complex macromolecules such as proteins, starch, and dietary fibers into smaller, more bioaccessible compounds, thereby enhancing the nutritional and functional attributes of fermented substrates [6,7]. Meng et al. [8] found that the content of cellulose, hemicellulose, and ligninin okara decreased from 24.87%, 21.56%, and 8.17% to 6.86%, 13.28%, and 6.51%, respectively; soluble dietary fiber (SDF) increased by 7.51 times after *B. subtilis* BSNK-5 fermentation. *B. subtilis* fermentation could increase the polyphenol content of highland barley bran by 203.17% [9]. Germinated and non-germinated brown rice fermented with *B. natto* presented an increased 2,2-diphenyl-1-picrylhydrazyl (DPPH) free radical scavenging and high nattokinase activity after *B. natto* fermentation [10]. Our previous study demonstrated that the content of cellulose, hemicellulose, and lignin in millet bran after *Bacillus subtilis* subsp. *natto* fermentation decreased (17.3% to 14.3%, 47.3% to 44.4%, and 8.8% to 7.9%, respectively); the SDF/insoluble dietary fiber (IDF) ratio has increased from 3.1% to 19.9% [11]. Fermentation of okara with *B. subtilis* DC-15 increased content of amino acids, dipeptides, fatty acids, small molecule sugars, and vitamins [12]. However, these studies mainly focused on changes in a single or a few components after fermentation, and the changes in microbial growth, enzyme activities, and the accumulation of these bioactive compounds with fermentation time remains inadequately explored. We hypothesized that there is a dynamic interplay among microorganisms, enzyme activities, and products, forming a sequential link that evolves over the course of fermentation.

Therefore, this study aims to investigate the effects of fermentation time on: (1) the growth dynamics of *B. natto*; (2) the activities of key enzymes (protease, amylase, cellulase, and NK fibrinolytic activity); and (3) the content of bioactive components including SDF, peptides, polyphenols, total sugars, reducing sugars, arabinoxylan, and β-glucan in millet bran. By analyzing these interconnected parameters, we seek to elucidate the underlying mechanisms governing the fermentation process and identify the optimal fermentation duration for producing millet bran extract with maximized bioactive compound content. These findings are expected to provide valuable insights into millet bran biotransformation and support its potential application as a functional food ingredient.

## 2. Materials and Methods

### 2.1. Materials

Millet bran was obtained as a processing by-product of “Huangjingu” from Xingtai City (Hebei, China). *Bacillus subtilis natto* (*B. natto*) was isolated and preserved by the Enzyme Engineering Laboratory of the Department of Bioengineering, Nanjing Agricultural University (Jiangsu, China). Congo red, Urokinase and β-glucan were purchased from Macklin Biochemical Technology Co., Ltd. (Shanghai, China). Folin-Ciocâlteu reagent was purchased from Sigma-Aldrich (Shanghai, China) Trading Co., Ltd. (Shanghai, China). Solarbio Science and Technology Co., Ltd. (Beijing, China) provided bovine fibrinogen. Casein was obtained from Sun Chemical Technology Co., Ltd. (Shanghai, China). Gallic acid was purchased from Aladdin Biochemical Technology Co., Ltd. (Shanghai, China). All other chemicals used were of analytical grade.

### 2.2. Fermentation of Millet Bran with B. natto

#### 2.2.1. Preparation of Defatted Millet Bran

Preparation of defatted millet bran was according to the method of Chu et al. [11] with some modification. Fresh millet bran was crushed and sieved through an 80-mesh sieve. It was then mixed with n-hexane at a ratio of 1:2.5 (g:mL) and subjected to defatting treatment using a magnetic stirrer at 55 °C. Each defatting cycle lasted 2 h. After completion, the supernatant was removed, and n-hexane was added again to the precipitate. This process was repeated three times in total. Upon completion of defatting, the precipitate was air-dried in a fume hood and then stored at −20 °C for later use.

#### 2.2.2. Fermentation of Millet Bran

To prepare the inoculum, *B. natto* preserved in a glycerol stock tube was inoculated onto a slant medium (peptone 1 g, beef extract 0.5 g, sodium chloride 0.5 g, agar 1.5 g, 100 mL distilled water) for activation. The activated culture was then transferred into Nutrient Broth (NB) medium (Peptone 1 g, beef extract 0.3 g, sodium chloride 0.5 g, 100 mL distilled water, pH 7.0–7.2), and incubated at 37 °C and 180 rpm until the culture reached the logarithmic growth phase (12 h). The bacterial suspension was adjusted to a concentration of 10^8^–10^9^ CFU/mL using sterile physiological saline (0.9%, *w*/*v*). Millet bran samples were fermented with *B. natto* following the procedures described by Yang et al. [13] with minor modifications. 10 g of defatted millet bran was mixed with 100 mL of distilled water containing 0.1 g glucose and 0.5 g sodium chloride and sterilized in an autoclave at 115 °C for 30 min. After cooling, 3% (*v*/*w*) of seed culture was inoculated into the millet bran medium, after thorough mixing, the inoculated millet bran was incubated in a shaking incubator at 37 °C and 180 rpm for 84 h. Samples were taken every 12 h. One portion of the fermentation broth was stored at 4 °C for viable bacterial count and spore count, while the other portion was centrifuged at 5000 rpm for 20 min and filtered through a 0.45 μm filter membrane to obtain the fermentation supernatant. One portion of the supernatant was stored at 4 °C as crude enzyme solution for enzyme activity assay, while the other portion was subjected to freeze-drying for the determination of bioactive components. The fermentation was conducted in triplicate for each millet bran sample.

### 2.3. B. natto Cell and Spore Count

2 mL of fermentation broth was taken and divided into two equal portions. One portion was used for *B. natto* cell count determination, while the other portion was treated in an 80 °C water bath for 15 min for spore counting. Briefly, A 0.1 mL aliquot of the appropriately diluted sample was spread onto Luria-Bertani (LB) agar plates, with triplicates prepared for each sample. All plates were inverted and incubated at 37 °C for 18–24 h, followed by colony enumeration [14,15]. Bacterial plate count was determined in accordance with GB 4789.2-2022 [16].

### 2.4. Enzyme Activity Assay

#### 2.4.1. Amylase Activity

Amylase activity was determined following the method of Ezeji et al. [17]. The crude enzyme solution and a 2% starch solution were pre-incubated in a 50 °C water bath. Then, 1.5 mL of the 2% starch solution was mixed with 0.5 mL of the crude enzyme solution (diluted 5×), vortexed thoroughly, and incubated at 50 °C for 30 min. The reaction was terminated by heating in a boiling water bath for 5 min. Subsequently, the reaction mixture was combined with 1.5 mL of 3,5-Dinitrosalicylic acid (DNS) reagent, mixed, heated in a boiling water bath for 5 min, and immediately cooled in an ice bath. The absorbance (OD540) of the mixture was measured. For the control group, the crude enzyme solution was first inactivated by heating in a boiling water bath for 5 min before reacting with the starch solution. One unit of amylase activity was defined as the amount of enzyme that can liberate 1 μg of glucose per minute from soluble starch under the assay conditions [18]. The glucose standard curve was: *y* = 1.2898*x* + 0.0207, R^2^ = 0.9973. (*y* is the absorbance value, *x* is the concentration of glucose in 0–1.0 mg/mL).

#### 2.4.2. Protease Activity

Protease activity was determined using Folin-phenol method [19]. One mL of crude enzyme solution was mixed with an equal volume of 1% casein solution, and incubated at 40 °C for 10 min. Then, 2 mL of trichloroacetic acid (TCA, 0.4 mol/L) was added to the system. It was allowed to stand for 10 min and then centrifuged at 5000 rpm for 20 min. A 30 μL aliquot of the supernatant was taken, and 140 μL of Na_2_CO_3_ (0.4 mol/L) and 30 μL of Folin reagent were added sequentially. After mixing, the mixture was incubated at 40 °C for 20 min. Following incubation, the absorbance at 660 nm was measured. For the control, 1 mL of crude enzyme solution was first mixed with an equal volume of TCA solution, then combined with the 1% casein solution. One unit of protease activity was defined as the amount of enzyme that can liberate 1 μg of tyrosine from casein per minute under the assay conditions. The tyrosine standard curve was: *y* = 0.0047*x* + 0.0917, *R*^2^ = 0.9979 (*y* is the absorbance value, *x* is the concentration of tyrosine in 0–70 μg/mL).

#### 2.4.3. Cellulase Activity

Cellulase activity was determined following the method of Zhang et al. [20]. The crude enzyme solution and a 0.5% sodium carboxymethyl cellulose (CMC-Na) solution in citric acid buffer (0.1 mol/L, pH 4.8) were pre-incubated separately in a 50 °C water bath. Subsequently, 1.5 mL of the CMC-Na solution and 0.5 mL of the crude enzyme solution were thoroughly mixed and incubated at 50 °C for 30 min. After the reaction, 1.5 mL of DNS reagent was added to the mixture, heated in a boiling water bath for 5 min, and then cooled in ice water. The OD540 was measured using a microplate reader. For control group, the crude enzyme solution was inactivated by heating in a boiling water bath for 5 min before mixing with the CMC-Na solution. One unit (1 U) of enzyme activity was defined as the amount of enzyme that can liberate 1 µg of glucose from CMC-Na per minute under the assay conditions.

#### 2.4.4. Fibrinolytic Activity of NK

Fibrinolytic activity of NK was determined using agarose-fibrin plate method according to the procedure of Xiong et al. [21]. One hundred mL of a 1% agarose solution was heated until completely transparent and then kept warm in a 50 °C water bath for 20 min. 75 mL of a 0.89 mg/mL fibrinogen solution was placed in a 50 °C water bath for 10 min. The agarose and fibrinogen solutions were then quickly mixed, and 5 mL of a 7.5 U/mL thrombin solution was added. The three components were thoroughly mixed and poured into a 9 cm diameter Petri dish. The dish was left at room temperature for 0.5 h to solidify, after which wells were punched for loading samples. 10 μL of the crude enzyme solution was added to the pre-punched wells on the agarose-fibrin plate, which was then placed in a 37 °C incubator for 18 h. The diameters of the dissolution zones were measured using a vernier caliper. A significant log-log linear relationship was observed between the product of the two perpendicular diameters of the lysis zones and the enzyme activity of the urokinase reference standard. A standard curve was constructed using urokinase (50–1600 U/mL) as the reference standard (*y* = 0.3305*x* + 1.4738, *R*^2^ = 0.9944), and the fibrinolytic activity of NK was subsequently calculated as its equivalent urokinase activity (U/mL), based on their ability to dissolve fibrin clots within the identical incubation period.

### 2.5. Determination of Total Sugar and Reducing Sugar Content

A 400 mg sample of dried fermented sample was dissolved in 25 mL of distilled water to prepare the test solution. Total sugar content was determined using the phenol-sulfuric acid method [22]. 5 mL of the test solution was placed in a stoppered test tube, and 5 mL of 6 mol/L HCl was added. The tube was sealed and placed in a 100 °C water bath for 30 min. Iodine solution was used to test the hydrolysis degree. Subsequently, 6 mol/L NaOH was used adjust the pH to neutral. Distilled water was added to bring the total volume to 25 mL, and the solution was vortexed to obtain the total sugar sample solution. This total sugar solution was diluted 40-fold with distilled water. One mL of the diluted solution was mixed with 0.5 mL of 5% phenol, followed by the addition of 2.5 mL of concentrated sulfuric acid. After mixing, the solution was kept at room temperature for 30 min, and the OD490 was measured using a microplate reader.

Reducing sugar content was determined using the DNS reagent method [23]. A 0.1 mL aliquot of the test solution (diluted 10-fold) was mixed with 0.3 mL of DNS reagent, heated in a boiling water bath for 5 min, and immediately cooled to room temperature under running water. Distilled water was added to bring the total volume to 1 mL. The OD540 was measured using a microplate reader, and the reducing sugar content in the sample was calculated based on the glucose standard curve (*y* = 1.2856*x* + 0.0449, *R*^2^ = 0.9995, concentration of glucose solution in 0–0.5 mg/mL).

### 2.6. Determination of β-Glucan and Arabinoxylan Content

β-Glucan content was determined using the Congo red spectroscopic method according to the procedure of Xia et al. [24] with slight modification. 1 g of the dried fermentation sample was dissolved in 30 mL of distilled water under sonication for 30 min. 2 mL of the sample solution was mixed with 4 mL of 0.01% Congo red solution by vortexing and then incubated at 20 °C. After 30 min, the absorbance at 545 nm was measured using a Varioskan Flash (Thermo Fisher Scientific, Vantaa, Finland). The β-glucan content in the dried fermentation sample was calculated according to the β-glucan (0–1.0 mg/mL) standard curve (*y* = 0.1475*x* + 0.1926, *R*^2^ = 0.9628).

Arabinoxylan content was determined using the phloroglucinol method [25]. A 400 mg of dried fermentation sample was dissolved in 25 mL of distilled water under sonication for 30 min. After complete dissolution, 2 mL of sample solution was taken and added to a stoppered tube, followed by the addition of 10 mL of reaction solution (2 mL concentrated hydrochloric acid, 2 g phloroglucinol, 10 mL anhydrous ethanol, 1 mL of 17.5 g/L glucose solution, and 110 mL glacial acetic acid). After vortex mixing, the mixture was placed in a boiling water bath for 25 min. Following the reaction, the mixture was cooled to room temperature in an ice bath. The absorbances at 552 nm and 510 nm were measured, respectively, and the difference was calculated. The arabinoxylan content in the sample was determined based on the xylose (0–1.0 mg/mL) standard curve (*y* = 0.1914*x* − 0.0159, *R*^2^ = 0.9866).

### 2.7. Determination of Polyphenol Content

For the determination of polyphenol content in fermented samples, the Folin–Ciocalteu method was employed, following the procedure reported by Boukhary et al. [26] with slight modifications. A 0.5 g dried sample was mixed with 10 mL of 80% methanol under ultrasonication treatment (Ultrasonic Cleaning Machine, Kun Shan Ultrasonic Instruments Co., Ltd., Kunshan, China). The mixture was then centrifuged at 5000 rpm for 20 min, and the supernatant was collected. The procedure was repeated two times and the supernatants were combined. The combined supernatant was adjusted to a final volume of 20 mL with 80% methanol to obtain the crude polyphenol extract. A 0.5 mL aliquot of the extract was sequentially mixed with 0.5 mL of Folin–Ciocalteu reagent and 1 mL of Na_2_CO_3_ solution (12.5%). Distilled water was added to bring the total volume to 5 mL. The mixture was vortexed and kept in the dark at room temperature for 80 min. Subsequently, the absorbance at 760 nm was measured. The results were expressed as micrograms of gallic acid equivalents per gram of millet bran (μg GAE/g MB). The gallic acid standard curve was: *y* = 0.0039*x* + 0.0439, *R*^2^ = 0.9946 (concentration of gallic acid in 0–100 μg/mL).

### 2.8. Determination of Polypeptide Content

Polypeptide content was determined using the biuret method, referring to the procedure reported by Wang et al. [27]. A 400 mg sample of dried fermented product was dissolved in 20 mL of distilled water by sonication for 30 min. After complete dissolution, 0.8 mL of the sample solution was mixed with an equal volume of 10% TCA (100 g L^−1^) and allowed to stand at room temperature for 30 min. The mixture was then centrifuged at 5000 rpm for 20 min. Then 1 mL of the supernatant was added to 4 mL of biuret reagent (0.375 g CuSO_4_·5H_2_O, 1.5 g KNaC_4_H_4_O_6_·4H_2_O, 75 mL of 10% NaOH, and 175 mL deionized water), mixed, and allowed to stand at room temperature for 30 min. The absorbance at 540 nm was measured, and the polypeptide content was calculated based on the bovine serum albumin (BSA) standard curve (*y* = 0.026*x* + 0.0735, *R*^2^ = 0.9978, concentration of BSA solution in 0–10 mg/mL).

### 2.9. Determination of SDF Content

The content of SDF was determined via the enzymatic–gravimetric method in accordance with the national standard GB 5009.88-2023 [28]. Briefly, 1 g dried sample from fermented or unfermented supernatant was totally dissolved in 10 mL water. The resulting solution was precipitated by the addition of four volumes of 95% (*v*/*v*) ethanol, and the mixture was stored overnight at 4 °C to ensure complete precipitation. Then the precipitated residue was collected by filtration, dried at 60 °C to constant weight, and the content of SDF was obtained.

### 2.10. Data Statistics and Analysis

All determinations were performed independently in triplicate and expressed as mean ± standard deviation (Mean ± SD). Data were subjected to significance analysis using SAS 9.4 software (SAS Institute Inc., Cary, NC, USA) and Duncan’s multiple range test, with *p* < 0.05 considered statistically significant. Graphs were plotted using OriginPro 2018 software developed and distributed by by OriginLab Corporation (Northampton, MA, USA).

## 3. Results

### 3.1. Dynamic Changes in the Number of Viable Bacteria and Spore During B. natto Fermentation

**Figure 1:**
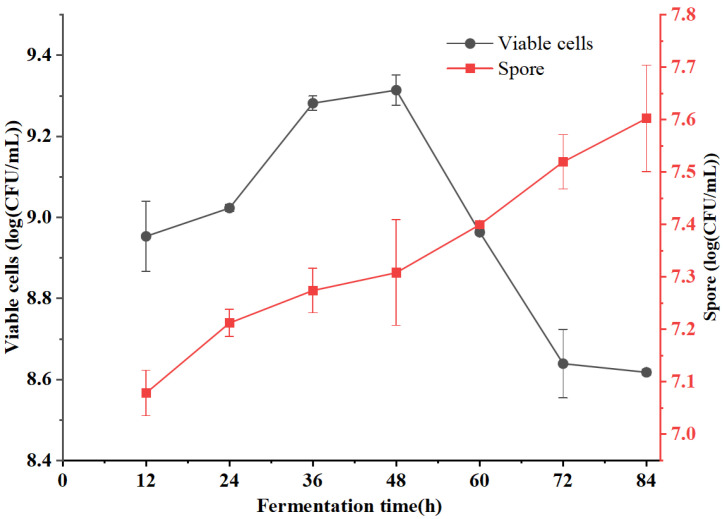
Dynamic changes in the viable bacterial count and spore count in millet bran during fermentation with *B. natto*.

The dynamic profile of viable cells and spores during the fermentation is presented in Figure 1. The viable cell count increased during the early and mid-stages, reaching a maximum of 9.3 log CFU/mL at 48 h, after which it gradually declined. Notably, the viable cell count remained consistently high, always exceeding 8.5 log CFU/mL throughout the entire fermentation period (0–84 h). Sporulation was observed at 12 h, which was consistent with the earlier formation of spores in *Bacillus subtilis* reported by Chen et al. [29], who reported that *B. subtilis* K-6-9 initiated spore formation at 9 h with a marked increase in spore count by 18 h. As the fermentation progressed and available nutrients were depleted, sporulation intensified, leading to a substantial increase in spore count, which ultimately reached 7.6 log CFU/mL by the end of fermentation (84 h).

### 3.2. Dynamic Changes in Different Enzyme Activities in Millet Bran During B. natto Fermentation

**Figure 2:**
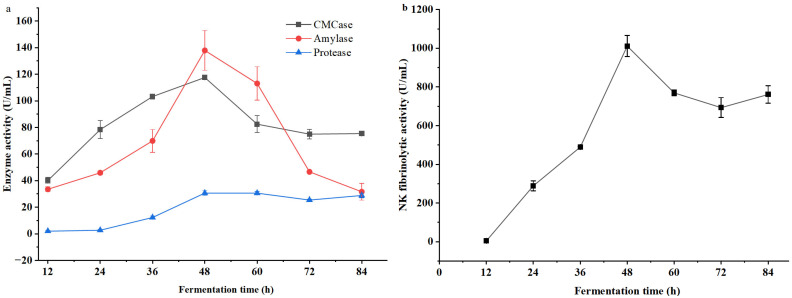
Dynamic changes in CMCase, protease and amylase activities (**a**) and nattokinase (NK) fibrinolytic activity (**b**) in millet bran during fermentation with *B. natto*.

The dynamic changes in the activities of protease, amylase, and cellulase during the fermentation of millet bran by *B. natto* are presented in Figure 2a. In the early fermentation phase (0–24 h), only a low level of protease activity was detected; after 24 h, protease activity increased gradually, peaking at 30.7 U/mL at 48 h before a slight decline, with the final activity reaching 14.16 times that observed at 12 h. Amylase activity rose rapidly within the first 48 h and then decreased sharply, with a maximum value of 137.99 U/mL at 48 h. CMCase activity exhibited a similar trend to amylase activity, attaining a peak of 117.7 U/mL at 48 h. The activity profiles of these three hydrolases were consistent with the variation in the viable cell count of *B. natto* (Figure 1). Notably, amylase activity started to increase rapidly at 12 h of fermentation, whereas CMCase activity showed a slow rise after this time point and the increment in protease activity was negligible compared to that of amylase and CMCase. Furthermore, the peak amylase activity (~140 U/mL) was significantly higher than the peak activities of CMCase (~120 U/mL) and protease (~30 U/mL), with all three enzymes reaching their maximum activities at approximately 48 h of fermentation.

The effect of fermentation time on the fibrinolytic activity of NK in the fermentation broth is shown in Figure 2b. NK fibrinolytic activity increased rapidly over the first 48 h, reaching a maximum of 1011.5 U/mL at 48 h. In the late fermentation phase (after 48 h), NK fibrinolytic activity decreased gradually with the extension of fermentation time. The dynamic change pattern of NK fibrinolytic activity was consistent with the growth of *B. natto*.

### 3.3. Effect of B. natto Fermentation on Total Sugar and Reducing Sugar Content

**Figure 3:**
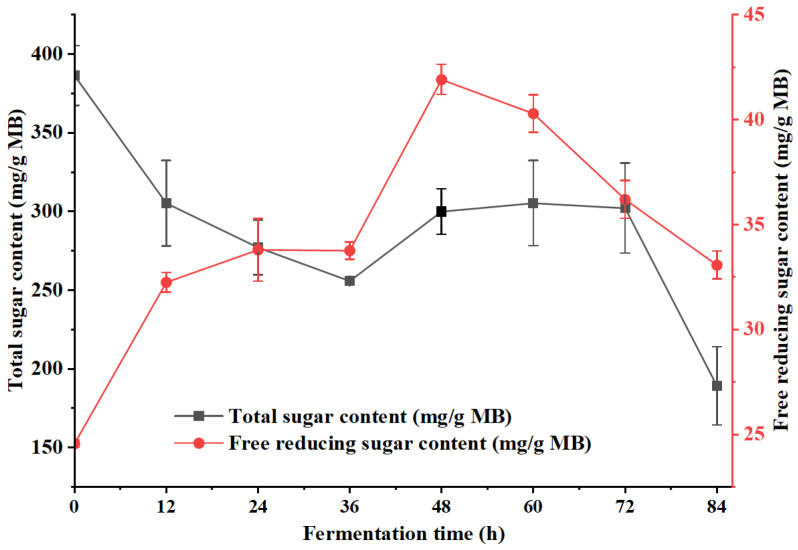
Dynamic changes in total sugar and free reducing sugar content in millet bran during fermentation with *B. natto*.

The dynamic changes in total sugar content and free reducing sugar content during *B. natto* fermentation of millet bran over 0–84 h is shown in Figure 3. At the start of fermentation (0 h), the total sugar content was relatively high (≈390 mg/g MB). It then decreased rapidly by 36 h, followed by a stable plateau (290–310 mg/g MB) between 48 h and 72 h. A second significant decline occurred after 72 h. In contrast to the trend observed for total sugar, the free reducing sugar content increased sharply in the initial 0–12 h, followed by slight fluctuations between 12 h and 36 h. It reached a peak of 41.9 mg/g MB at 48 h, then gradually decreased.

### 3.4. Dynamic Changes in Bioactive Component Contents in Millet Bran During Fermentation with B. natto

#### 3.4.1. Polypeptide Content

**Figure 4:**
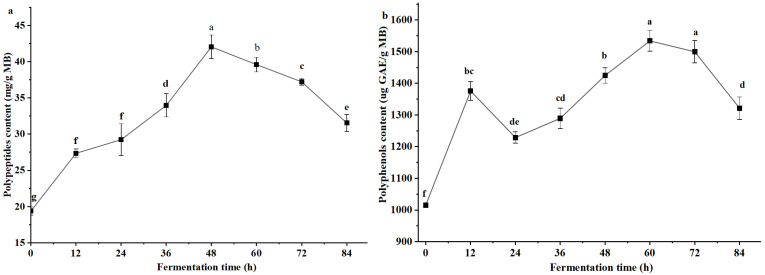
Dynamic changes in polypeptides content (**a**) and polyphenols content (**b**) in millet bran during fermentation with *B. natto.* Different lowercase superscript letters indicate significant statistical differences among groups (*p* < 0.05).

The dynamic changes in polypeptide content in millet bran over the course of fermentation are presented in Figure 4a. Polypeptide content increased significantly with fermentation time, peaking at 42.0 mg/g MB at 48 h, and then gradually decreased thereafter. This trend was consistent with the changes in protease activity observed in Figure 2. Compared with unfermented millet bran, the polypeptide content in millet bran fermented for 48 h was increased by 116.8%.

#### 3.4.2. Polyphenol Content

Dynamic changes in polyphenol content in millet bran across different fermentation time points are shown in Figure 4b. During fermentation, the polyphenol content in millet bran exhibited a general trend of initial increase followed by decrease with prolonged fermentation time. The highest polyphenol content (1534.1 μg GAE/g MB) was achieved at 60 h of fermentation. This level was 1.5 times greater than that in unfermented millet bran (0 h).

#### 3.4.3. Dynamic Changes in SDF Content in Millet Bran During *B. natto* Fermentation

**Figure 5:**
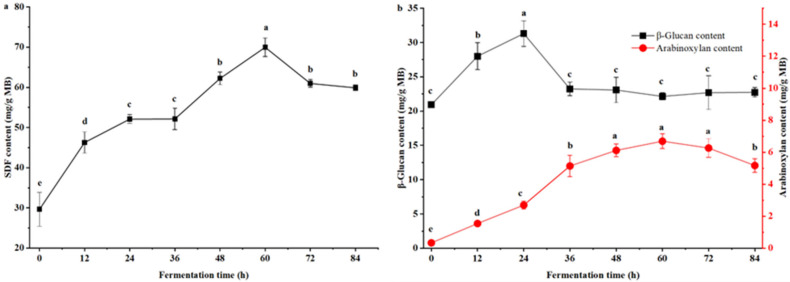
Dynamic changes in soluble dietary fiber (SDF) (**a**) and polysaccharides (**b**) content in millet bran during fermentation with *B. natto*. Different lowercase superscript letters indicate significant statistical differences among groups (*p* < 0.05).

The effect of fermentation time on SDF content in millet bran is presented in Figure 5a. From 0 to 60 h, the SDF content increased gradually with prolonged fermentation time, whereas further extension of fermentation led to a subsequent decline. The SDF content reached its maximum at 60 h, with a value of approximately 70.0 mg/g MB, which was 2.4-fold higher than that of unfermented millet bran.

#### 3.4.4. Dynamic Changes in β-Glucan and Arabinoxylan Content in Millet Bran During *B. natto* Fermentation

The effect of fermentation time on the contents of β-glucan and arabinoxylan in millet bran is presented in Figure 5b. During the 0–24 h fermentation phase, β-glucan content increased gradually, reaching a maximum of 31.31 mg/g MB at 24 h. Subsequently, it first decreased and then stabilized, with a content of 22.16 mg/g MB at 60 h.

With the extension of *B. natto* fermentation time, arabinoxylan content increased progressively, reaching a maximum of 6.69 mg/g MB at 60 h—19.68-fold that of unfermented millet bran (0.34 mg/g MB). Further prolongation of fermentation time led to a gradual decline in arabinoxylan content.

## 4. Discussion

This study systematically reveals the synergistic dynamics among microbial growth, enzymatic activity, and the generation of multiple active components during the fermentation of millet bran by *B. natto*. These results will provide a scientific basis for optimizing fermentation processes and developing high-value-added millet bran products.

### 4.1. Interrelationship Between Microbial Growth and Metabolic Progression

The fermentation process exhibited characteristic growth-associated metabolic kinetics [30,31], The viable cell counts of *B. natto* increased rapidly, reaching a peak of 9.31 log CFU/mL at 48 h (Figure 1). Notably, the activities of protease, amylase, cellulase, and NK fibrinolytic activity were synchronized with microbial growth, all reaching maximum levels at the same time point (Figure 2). This strong synchrony indicates that the synthesis of these hydrolytic enzymes and NK is integral to the primary metabolism of *B. natto*, primarily serving to decompose macromolecules (proteins, starch, cellulose) in the millet bran to acquire nutrients and energy for growth.

After 48 h, following the rapid depletion of nutrient substrates (particularly total sugars; Figure 3), *B. natto* transitioned from the exponential phase to the late stationary phase and initiated sporulation [32]. As an obligate aerobe, the rapid proliferation and robust metabolism of *B. natto* likely induced environmental stressors such as oxygen limitation, further promoting spore formation [33,34]. As sporulation involves the differentiation of vegetative cells into dormant spores [35], the reduction in metabolically active vegetative cells led to diminished enzymatic synthesis. Additionally, the decline in enzyme activity after 48 h may be attributed to the degradation of vegetative-phase enzymes, inactivation of biosynthetic pathways, or autodegradation [36,37].

The dynamic profiles of extracellular enzymes (Figure 2a) further revealed *B. natto*’s metabolic adaptation and substrate utilization strategy. Amylase, CMCase, and protease all exhibited a synchronized peak at 48 h, but differed in their activation rates and maximum activities. Amylase activity increased rapidly after 12 h to a peak of 137.99 U/mL, which was significantly higher than CMCase (117.7 U/mL) and protease (30.7 U/mL). This discrepancy reflects the strain’s metabolic priority—*B. natto* preferentially utilizes easily degradable starch first. The relatively high cellulase activity was ascribed to the inductive action of the abundant lignocellulose in millet bran [38,39]. This substrate-dependent enzyme regulation is a typical adaptive mechanism for microorganisms to maximize nutrient utilization efficiency in complex agricultural byproducts [20].

Consistent with the enzyme activity profiles, reducing sugar content peaked at 48 h (41.92 mg/g MB, Figure 3), which might be a result of starch and cellulose hydrolysis by amylase and cellulase secreted during *B. natto* growth [40]. At this time point, the rate of reducing sugar generation from macromolecular substrate hydrolysis might exceed microbial consumption for growth and maintenance, forming a transient “sugar reservoir” [41]. This abundant carbon source supported the highest level of metabolic activity and enzyme synthesis, explaining why microbial counts, enzyme activities, and reducing sugar content all coincided at 48 h as the metabolic peak of fermentation.

### 4.2. Differential Kinetics and Mechanistic Insights into the Generation of Active Components

The accumulation of different bioactive components followed distinct temporal kinetics, reflecting the diversity of their biosynthetic pathways. Polypeptide content (Figure 4a) closely mirrored the protease activity profile (Figure 2a), both peaking at 48 h. This provides direct evidence that polypeptides are immediate products of protease-mediated hydrolysis of millet bran proteins, whose accumulation is tightly regulated by protease activity [42]. In contrast, polyphenols and SDF reached their maximum levels at 60 h (Figure 4b and Figure 5a), lagging behind the peak of primary metabolism (48 h). This delay suggests their generation involves more complex mechanism. These components are likely released or converted by the prolonged action of cell wall-degrading enzymes (amylase, cellulase, protease) secreted during the early metabolic phase. These enzymes gradually disrupt the millet bran matrix, releasing bound polyphenols and converting IDF to SDF. This observation is consistent with previous studies: Ren, Wang and Liu [43] reported positive correlations between amylase/protease activities and SDF, total phenolic content (TPC), and flavonoid levels during *Rhizopus oryzae* fermentation of wheat bran; Qin et al. [44] also found that phenolic acid release lagged behind β-glucosidase activity during lactic acid bacteria fermentation of passion fruit peel, attributed to the time-dependent breakdown of plant cell walls. A limitation of the current study is the sole determination of total phenolic content; future work will employ High Performance Liquid Chromatography (HPLC) to identify and quantify individual phenolic compounds, and further evaluate the antioxidant activity of fermented products.

The structural transformation of millet bran polysaccharides (β-glucan and arabinoxylan) further supported the enzymatic remodeling of substrates during fermentation. Notably, the arabinoxylan content increased 19.4-fold within 60 h (Figure 5b), indicating that its release from the bran matrix (due to cell wall disruption and substrate swelling) occurred faster than its microbial consumption. This “net accumulation” is supported by the observed significant positive correlation between arabinoxylan and free reducing sugar contents (r_s_ = 0.83, *p* < 0.05), which rules it out as a major degraded source for sugar accumulation. Conversely, β-glucan content showed no significant correlation with total sugar (r_s_ = −0.55, *p* = 0.16), underscoring that sugar dynamics were regulated by the hydrolysis of multiple polysaccharides (like starch, cellulose) alongside microbial metabolism, rather than by β-glucan degradation alone. Collectively, the enzymatic arsenal of *B. natto* effectively remodeled the fiber components, enhancing the bioavailability and functional properties of millet bran.

### 4.3. Implications for Process Optimization and Application Prospects

The dynamic profiles of fermentation indicators provide a precise roadmap for target-oriented process control. For maximizing enzymatic activities, especially for NK fibrinolytic activity or polypeptide yield, 48 h is the optimal fermentation endpoint, corresponding to the metabolic peak of *B. natto*. For maximizing antioxidant polyphenols or SDF production, fermentation should be extended to 60 h to allow sufficient time for enzyme-mediated release of bound components from the millet bran matrix. All these products can be used as a functional food ingredient for food industry.

The metabolic decline after 48 h might be closely linked to two factors: nutrient exhaustion and sporulation [45]. To address this, future studies could explore fed-batch fermentation strategies, such as supplementing limited readily available carbon/nitrogen sources around 48 h. This approach would sustain microbial metabolic activity, delay sporulation, and potentially extend the production plateau or enhance target product yields. Additionally, complementing total phenolic content with individual phenolic compound profiling (via HPLC) and evaluating their antioxidant activities will provide a more comprehensive understanding of the functional value of fermented millet bran.

## 5. Conclusions

In summary, fermentation with *B. natto* constitutes an efficient and versatile biotransformation strategy for the valorization of millet bran. A 48–60 h fermentation process enabled the synergistic biosynthesis of diverse high-value bioactive products, including functional enzymes (nattokinase), bioactive peptides, antioxidant polyphenols, and prebiotic dietary fibers (soluble dietary fiber, β-glucan, and arabinoxylan). This biotransformation not only markedly elevates the comprehensive nutritional and functional value of millet bran but also unlocks novel application avenues for it in the development of functional foods, nutritional supplements, and even pharmaceutical ingredients. While the present study elucidates the dynamic metabolic characteristics of *B. natto*-mediated millet bran fermentation and provides valuable scientific insights for its industrial utilization, it has notable limitations. The analysis of bioactive components (polyphenols, peptides, and soluble dietary fiber) was confined primarily to quantitative determination of their contents. In-depth qualitative characterization—including the identification of specific generated peptide sequences, profiling of individual polyphenol compounds, and analysis of the molecular weight distribution and structural modifications of SDF and arabinoxylan—would yield more profound insights into the functional quality and potential health-promoting effects of the fermented millet bran. Building on the findings of this study, subsequent investigations will focus on the aforementioned research gaps to fully exploit the functional potential of fermented millet bran.

## Data Availability

The original contributions presented in the study are included in the article, further inquiries can be directed to the corresponding author.
